# Compartments of the crural fascia: clinically relevant ultrasound, anatomical and histological findings

**DOI:** 10.1007/s00276-023-03242-4

**Published:** 2023-10-09

**Authors:** S. Ortiz-Miguel, M. Miguel-Pérez, J. Blasi, A. Pérez-Bellmunt, J. C. Ortiz-Sagristà, I. Möller, J. L. Agullo, P. Iglesias, C. Martinoli

**Affiliations:** 1 Unit of Human Anatomy and Embryology, Department of Pathology and Experimental Therapeutics, Faculty of Medicine and Health Sciences (Bellvitge Campus), University of Barcelona, C/Feixa Llarga, s/n, 08907 L′Hospitalet de Llobregat, Spain; 2 Basic Sciences Department, Universitat Internacional de Catalunya, Sant Cugat del Vallès (Barcelona), Spain; 3 Unit of Histology, Department of Pathology and Experimental Therapeutics, Faculty of Medicine and Health Sciences (Bellvitge Campus), University of Barcelona, L′Hospitalet de Llobregat, Spain; 4 Anesthesia Department, Fundació Puigvert, Barcelona, Spain; 5 Cattedra di Radiologia “R”-DICMI, Universita di Genova, Genoa, Italy

**Keywords:** Fascia, Ultrasound, Anatomical and histological study, Lower extremity, Implications

## Abstract

**Purpose:**

Compartment syndrome is a surgical emergency that can occur in any part of the body and can cause cell necrosis when maintained over time. The resulting defects can affect the nerves, muscle cells, bone tissue, and other connective tissues inside the compartment, and fasciotomy has to be performed. The anatomical and histological characteristics of the leg make acute, chronic, and exertional compartment syndrome more likely in this limb. For these reasons, knowledge of the ultrasound, anatomical, and histological features of the crural fascia can help in the treatment of leg compartment syndrome.

**Methods:**

Twenty-one cryopreserved lower limbs from adult cadavers and from one 29-week-old fetus were obtained from the dissection room. They were examined by ultrasound and a subsequent anatomical dissection and microscopy to study the crural fascia and its relationship with the different muscles. Anthropometric measurements were taken of the distances from the head of the fibula and lateral malleolus to the origin of the tibialis anterior muscle in the crural fascia, the exit of the superficial fibular nerve, and the fascia covering the deep posterior muscles of the leg.

**Results:**

The crural fascia has very important clinical relationships, which can be identified by ultrasound, as the origin of the tibialis anterior muscle at 16.25 cm from the head of the fibula and the exit of the superficial fibular nerve that crosses this fascia at 21.25 cm from the head of the fibula. Furthermore, the presence of a septum that fixes the deep posterior muscles of the leg and the vessels and nerve can be seen by ultrasound and can explain the possible development of a posterior compartmental syndrome of the leg. Awareness of these features will help to keep these structures safe during the surgical treatment of compartment syndrome.

**Conclusion:**

The ultrasound study allows identification of anatomical structures in the leg and, thus, avoids damage to them during surgery for compartmental syndromes.

## Introduction

Compartment syndrome is considered as a clinical emergency when there is a loss of perfusion in an extremity. It can appear in any part of the body containing myofascial compartments such as the upper limbs, thighs, abdomen, and buttocks [24]. However, it is more common in the leg and forearm, due to the large mass in the area and the inelasticity of the fascia [15].

In the leg, acute compartment syndrome is often caused by knee fractures, dislocations, tibial fractures, or soft-tissue injuries [13]. If left untreated, it will result in cellular anoxia [24]. Chronic compartment syndrome can be caused by the combination of increase in muscle volume during exercise [19] and the long-term presence of fibrosis, which increases the stiffness of the deep fascia. This crural fascia is composed of dense connective tissue and surrounds all the muscles of the leg. It also forms septa to compartmentalize the different groups of muscles [4].

These different layers can be seen by ultrasound, which can aid in diagnosis and application of different treatments [16]. The deep fascia is tougher in the back and lower limbs, where it forms dense connective tissue sheets with a large number of closely packed collagen fibers to adapt to the mechanical requirements of the region [21]. However, despite the advantages offered by the deep fascia, there are certain circumstances in which the thickness and stiffness of the fascia can compromise the tissue, including acute [17] and chronic compartment syndrome [6]. In the leg, the crural fascia appears as a lamina of connective tissue with a mean thickness of 0.650 mm, composed of three or, at some points, two layers of parallel collagen fiber bundles that are separated by a thin layer of loose connective tissue [3]. The connections between the muscles and muscular fasciae are constant and show precise organization [23]. Depending on the type of movement, specific muscles are activated, stretching selective portions of the muscular fascia through the actions of specific myofascial expansions. This organization can be observed in all the limbs, indicating that the fasciae act like a transmission belt between two adjacent joints and also between synergist muscle groups, guaranteeing perceptive and directional continuity and probably representing the anatomical basis of myokinetic chains [22].

Once compartment syndrome is diagnosed, it can be difficult to perform a fasciotomy without damaging certain anatomical structures such as the superficial fibular nerve [12]. This is why some studies believe that the ultrasound approach should be used to avoid iatrogenic damage [2]. However, anatomical structures present variations and may sometimes be difficult to recognize in an ultrasound study. The aim of this study was broadening our knowledge of the crural fascia and its anatomical relationships by performing an ultrasound, anatomical, and histological study. This information may help to guide the surgical treatment of compartment syndrome.

## Materials and methods

An observational ultrasound, anatomical, and histological study was performed on a total of 21 lower limbs (9 left and 12 right) obtained from the cadavers of 10 women (44%) and 11 men (56%), ranging in age from 61 to 92 years (Table 1), as well as on one 29-week-old fetus. The limbs had been cryopreserved at −20 ºC in the dissection room of the Faculty of Medicine and Health Sciences at the University of Barcelona. They were numbered in order according to their ultrasound, anatomical, and histological study. Dysmorphic limbs and those that had undergone surgery or trauma in the area being studied were rejected and are not included in the study. The study was approved by the local ethics committee.

**Table 1:** Descriptive statistics, qualitative variables (number of de cadavers studied 12)

Variable	Category	*N* (percentage)
Sex	Man	7 (58.3%)
	Woman	5 (41.7%)
Side	Right	8 (66.7%)
	Left	4 (33.3%)

Prior to the study, one fresh lower limb was injected with black latex in the femoral artery and dissected in the anatomical position to observe the relationships of the crural fascia with the different muscles in the anterior, posterior, medial, and lateral compartments, and to visualize the different subcompartments that this fascia forms in the posterior compartment of the leg. The study began once the presence of these muscular subcompartments and anatomical structures had been confirmed.

### Ultrasound study

The ultrasound study was performed with the LOGIQ P9 ultrasound system (GE Ultrasound Korea LTD, Seongnam, Korea) with a high-frequency linear probe (6–15 MHz). First, in the transverse and longitudinal axis, we visualized the muscles of the leg compartments and their relationships with the crural fascia covering them.

To identify and study the crural fascia and its relationships with the muscles, we divided the lower limbs into two groups. In the first group containing 9 lower limbs and the 29-week-old fetus, an ultrasound-guided injection with a 20G needle was performed parallel to the transducer below the fascia covering the tibialis anterior muscle (TA) and the extensor digitorum longus muscle (EDL). This injection contained 2 ml of dye (0.5 ml of a red or green dye (Tempera mate arts Titan, manufacturastitan arts SLU. Sant Fruitos del Bages. Barcelona) and 1.5 mL of saline. For the lateral compartment, we visualized the fascia that covered the peroneus longus muscle (PL) and injected below it, also with ultrasound guidance. The probe was then moved to the posterior compartment and the same system was used, injecting the dye under the soleus to look for the compartment of the posterior deep muscles of the leg. In the short axis, a sweep was performed to confirm adequate diffusion of the dye inside the injected compartments.

In the second group containing 12 lower limbs, we took anthropometric measurements of the distances from the head of the fibula and lateral malleolus to the origin of the TA in the crural fascia, the exit of the superficial fibular nerve, and the fascia covering the deep posterior muscles of the leg.

### Anatomical study

After the ultrasound study, we performed a gross anatomical study by dissecting 17 legs, performing four transverse cuts on the leg at the point of the injection.

The anatomical dissection was first performed on the lateral side of the leg, following a classical method along the planes. A longitudinal lateral incision and two horizontal incisions were performed under the patella proximally and at the ankle distally. The posterior compartment was dissected in the same way.

In the first group, after dissection in the planes of the skin and subcutaneous or adipose tissue, the crural fascia of the anterior compartment was exposed and the location of the dye was studied to determine whether there had been any extravasation. The crural fascia was then cut 3 cm laterally from the anterior border of the tibia to avoid cutting the muscle fibers of the TA in this fascia. Subsequently, we observed the position of the dye for each of the muscles studied (TA and EDL). The same was undertaken in the lateral compartment (PL). In the posterior compartment, the crural fascia was cut along the posterior median line. The gastrocnemius and soleus muscles were identified and removed. When the deep posterior muscles were exposed, we checked that the dye was contained between the fasciae covering these muscles. On four legs, a sectional anatomy was undertaken by performing transverse cuts at the superior and inferior third of the leg to observe whether the dye was located below the fascia. This information was used to assess the relationship of the fascia with the muscles studied.

In the second group containing 12 lower limbs, we took anthropometric measurements of the distances from the head of the fibula and lateral malleolus to the origin of the TA in the crural fascia, the exit of the superficial fibular nerve, and the fascia covering the deep posterior muscles of the leg. The ultrasound and anatomical measurements were compared.

The leg of the fetus was dissected, and the dye was observed in the different compartments. At each step, images were taken to record information (Canon EOS 60D).

### Histological study

In one specimen, six different samples of a similar size (1 cm × 2 cm) were taken from different points of the crural fascia:

- in the superior third of the leg where the TA originates at the crural fascia.
- in the middle third of the anterior side of the leg.
- in the superior retinaculum above the TA and EDL.
- at the fascia covering the deep posterior muscles in the middle third of the posterior side of the leg.
- superficial to the gastrocnemius in the superior and inferior third of the crural fascia.

The samples were fixed in 4% formaldehyde and processed into paraffin blocks. They were then cut into 4 µm sections and dyed with the haematoxylin–eosin stain. The thicknesses of the fascia were measured and observed with a Leica Digital Microimaging Device (Leica DMD108 microscope), Fluorescent images were obtained from haematoxylin–eosin-stained sections using a Carl Zeiss Axioimager M2 (excitation 385/30 and emission 425/30). Fluorescence images allowed a better analysis of fascia organization into layers.

Statistical analysis was performed on all the data obtained for the control variables (sex, limb side, and age) and the anthropometric variables. The qualitative variables are presented in the form of absolute and relative frequency; whereas, the quantitative variables are presented in the form of median and interquartile range (IQR) and average with standard deviation. The agreement between the measures of the anatomical structures obtained by dissection and their estimation based on the measurement by ultrasound is studied by the coefficient intraclass correlation (ICC) and the Bland–Altman graphic.

## Results

### Ultrasound study

In all the lower limbs, the ultrasound study allowed the identification of the crural fascia, below the adipose tissue, as a continuous well-differentiated echogenic line surrounding the muscles of the anterior, posterior and lateral compartments and isolating each of these compartments. This information allowed the injection of the dye into each of the spaces below the fascia and the different muscles.

**Fig. 1:**
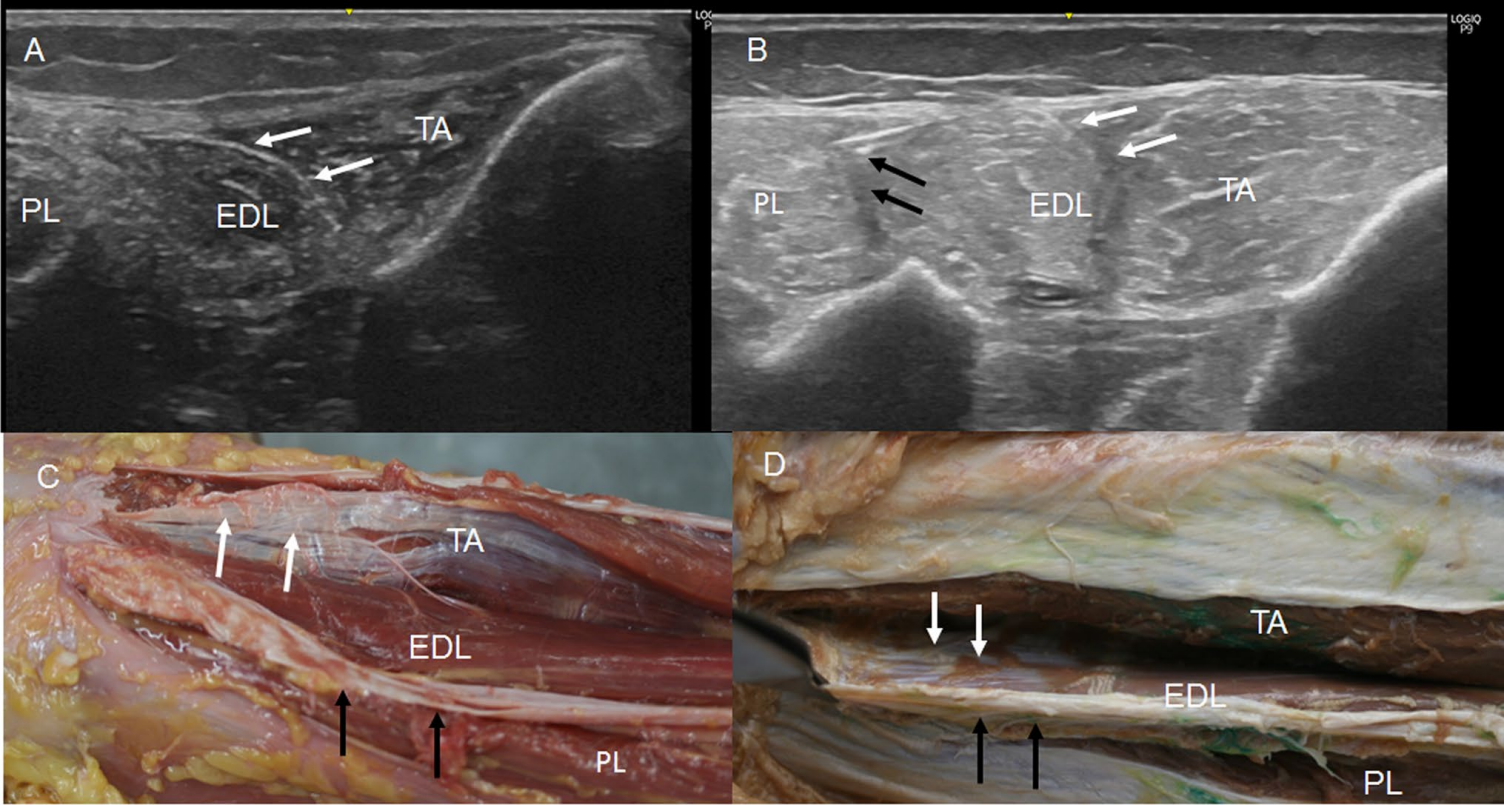
Ultrasound image of the septum between the tibialis anterior muscle (TA) and extensor digitorum longus muscle (EDL) A a hyperechoic line (white arrows) B a hypoechogenicity line (white arrows) between TA and EDL. Black arrows show the septum between the EDL and peroneus longus muscle (PL) C The dissection shows this septum (white arrows) as an aponeurosis that belongs to the TA. Black arrows show the septum between the EDL and PL . D It shows the aponeurosis that corresponds to the EDL. Black arrows show the septum between EDL and PL

**Fig. 2:**
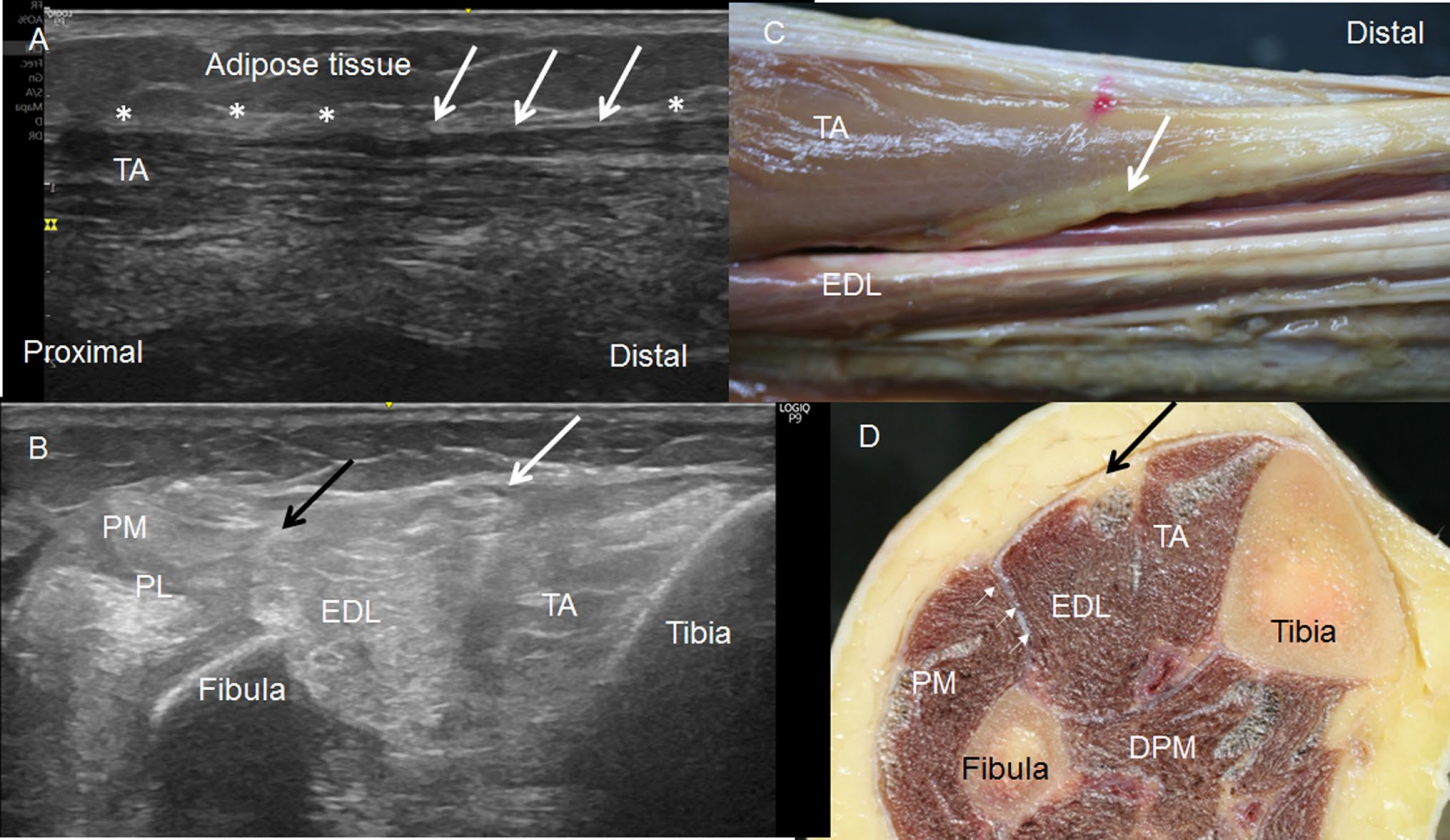
**A** Long-axis ultrasound view of the hypoechogenic image (white arrows) This area marks the end of the tibialis anterior muscle (TA) at the crural fascia (white asterisks).** B**: Short-axis ultrasound view of the leg at the middle third shows this hypoechogenicity image (white arrow) between TA and EDL. Laterally, the peroneus longus muscle (PL) is separated by a septum (black arrow) from the EDL.** C**: Anatomical study shows that the hypoechogenicity image corresponds to adipose tissue (white arrow) between TA and EDLof a right leg. D Transversal cut of the leg, at the middle third, shows the adipose tissue (black arrow) between the TA and EDL. Laterally, the peroneus muscles (PL) are separated from the EDL by a septum (short white arrows). Posteriorly the deep posterior muscles of the leg (DPM)

**Fig. 3:**
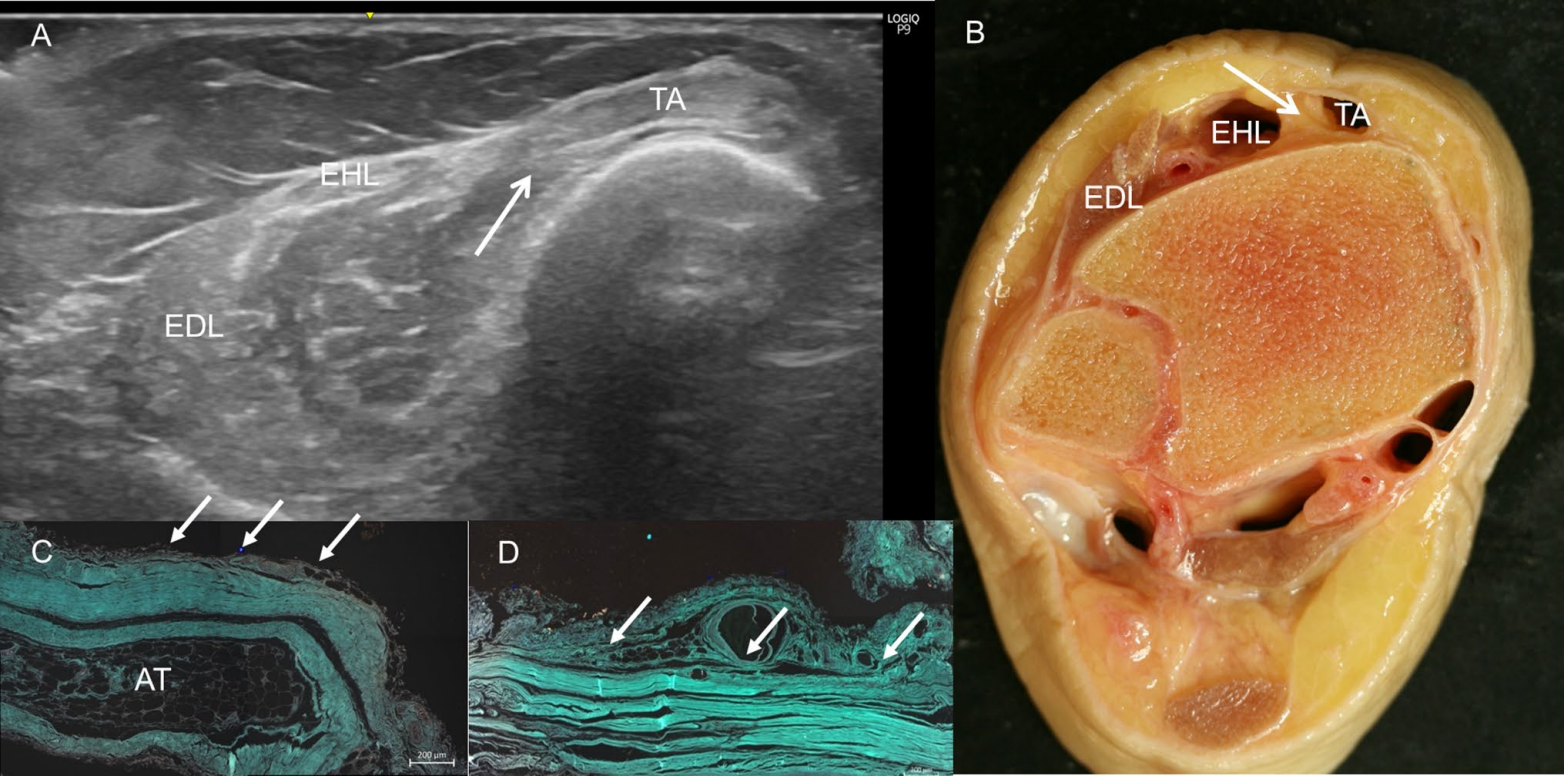
**A** An ultrasound view of the superior extensor retinaculum and an extension of it to the tibia to separate the tendon of the tibialis anterior muscle (TA) (white arrow) from the extensor hallucis longus muscle (EHL). Extensor digitorum longus muscle (EDL).** B** Anatomic view of these compartments without the tendons. The expansion of the superior extensor retinaculum arrives to the tibia (white arrow).** C** Histology image of the superior extensor retinaculum covering the tendon of the EDL (white arrows) with an adipose tissue under the retinaculum (AT)** D** Histological view of this retinaculum covering the tendon of the TA (white arrows). Notice the difference of the layers

A more detailed study of the crural fascia showed that in the anterior compartment, it gives a hyperechogenic or hypoechogenic line (depending on the perpendicular situation of the prove on the leg) between the TA and EDL that separated the two muscles, (Fig. 1A, B). Inferiorly, along the long axis, a hypoechogenic image (Fig. 2A), 16.25 cm from the head of the fibula marked the end of the TA at this fascia. It was also seen along the transversal axis, between the TA and EDL that distinguished these muscles from one another (Fig. 2B) Inferiorly, before the level of the ankle, the deep fascia made extensions into the bone to separate the tendon of the TA (Fig. 3A), from extensor hallucis longus muscle (EHL) and EDL. It was fixed at the medial malleolus and lateral malleolus medially and laterally, respectively.

**Fig. 4:**
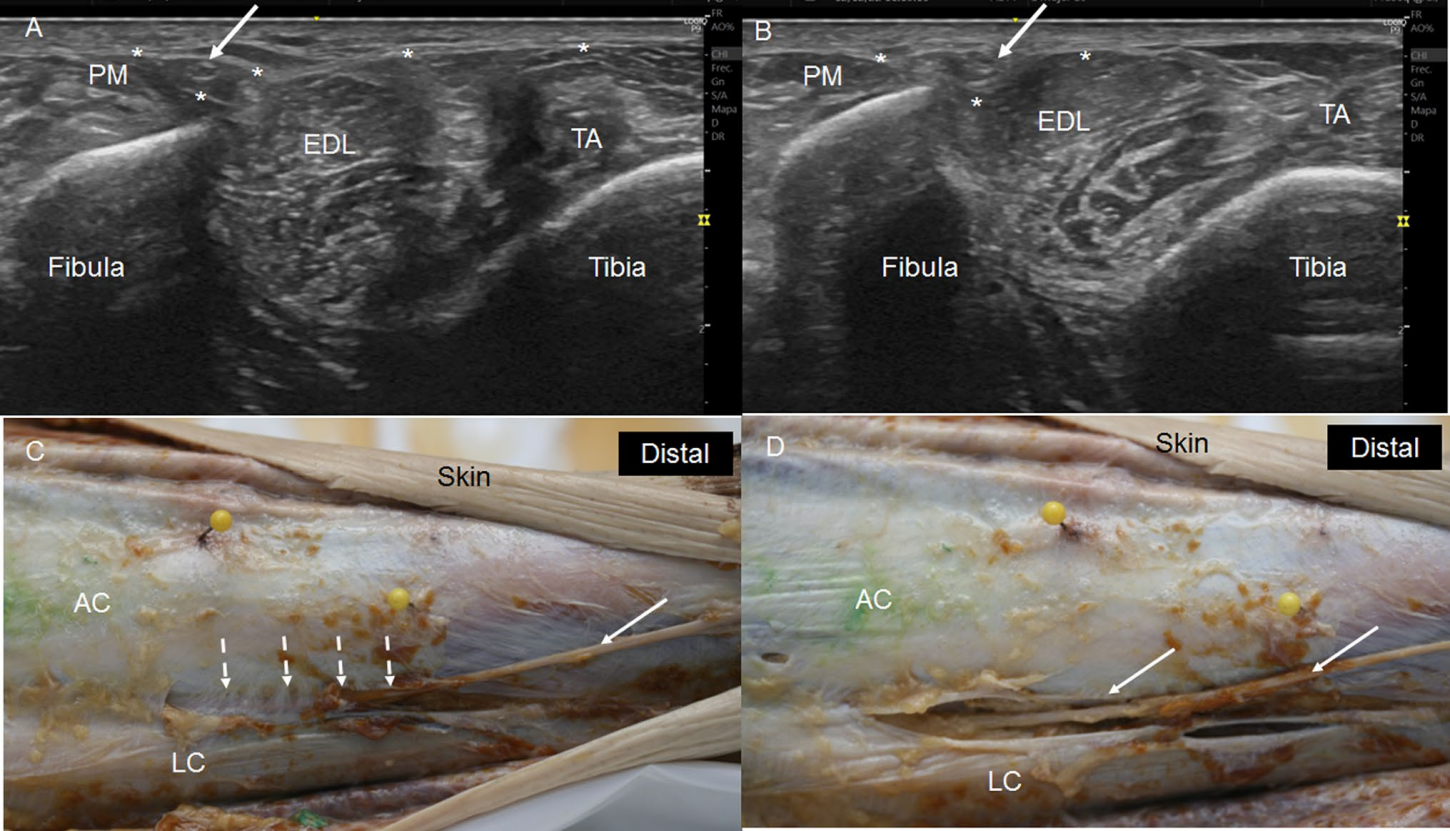
The exit of the superficial fibular nerve (SFN).** A** Ultrasound view of the SFN (white arrow) inside the tunnel that crural fascia (white asterisk) makes for the nerve, medially to the peroneus muscles (PM). Extensor digitorum longus (EDL) and tibialis anterior (TA) muscles covered by the crural fascia (white asterisks)** B** Ultrasound view of the exit of SFN (white arrow) after crossing the crural fascia (white asterisk).** C** Dissection of the leg shows the crural fascia under the skin forming the anterior (AC) and the lateral (LC) compartment of the leg. Yellow needles, medially located, and placed thanks to the previous ultrasound study, mark the tunnel under the crural fascia for the SFN (white intermittent arrows)** D** Exposition of the SFN inside the tunnel (white arrows), after cutting the crural fascia

In the lateral compartment, we observed the crural fascia fixed at the medial crest and lateral surface of the fibula, isolating the peroneus longus and peroneus brevis muscles. In this compartment, we found the superficial fibular nerve. This nerve can have a variable pathway before crossing the crural fascia but it finished crossing this fascia to exit from this compartment at 21.25 cm from the head of the fibula and 12.5 cm from the lateral malleolus. However, certain variations were observed. In one case, the nerve crossed the septum between the lateral and anterior compartments and continued in this anterior compartment until the exit. In another case, the nerve crossed the septum between the lateral and anterior compartments and returned to the lateral compartment before exiting definitively. Finally, in three of the cases studied (25%), the nerve crossed the crural fascia, but there was a tunnel formed by this fascia measuring 4.33 cm inside which the nerve was located surrounded by adipose tissue (Fig. 4A, B).

**Fig. 5:**
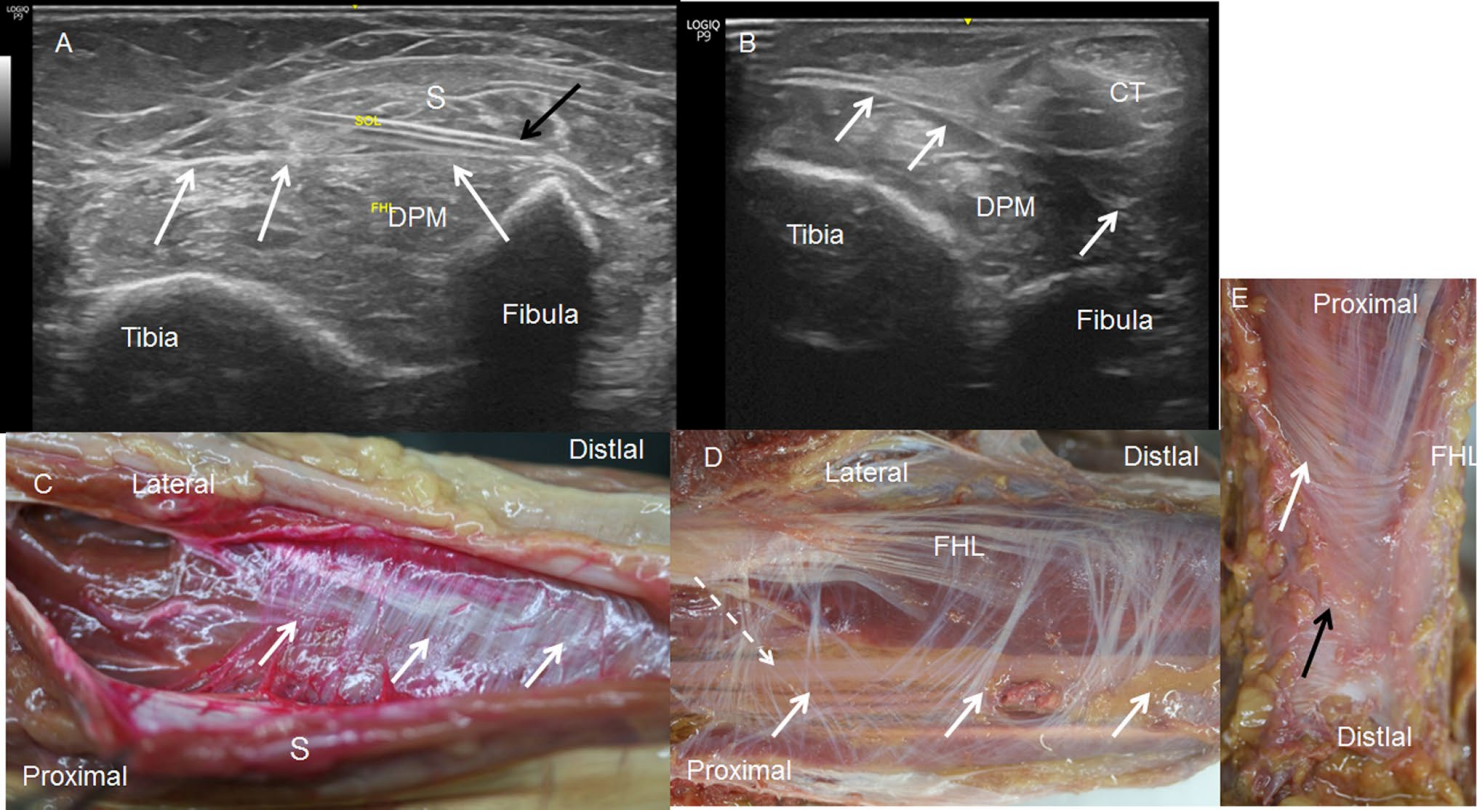
Posterior compartment of leg.** A** Ultrasound view of a right leg where the hyperechogenic line (white arrows) divided the deep posterior muscles of the leg (DPM) from the soleus muscle (S) The needle (black arrow) was under the soleus muscle to inject the dye.** B** The ultrasound view of the hyperechogenic line (white arrows) between the DPM and the calcaneal tendon (CT) at the inferior third of a left leg.** C**,** D**,** E** Anatomical view of the septum after the triceps surae muscle has been removed in all the examples. C Septum (white arrows) separates the deep posterior muscles of the leg from the soleus muscle (S).** D** Under the septum flexor hallucis longus (FHL) and tibialis nerve (white arrows) are identified.** E** The septum (white arrow) is thicker and more evident in the inferior part (black arrow) of the leg

**Fig. 6:**
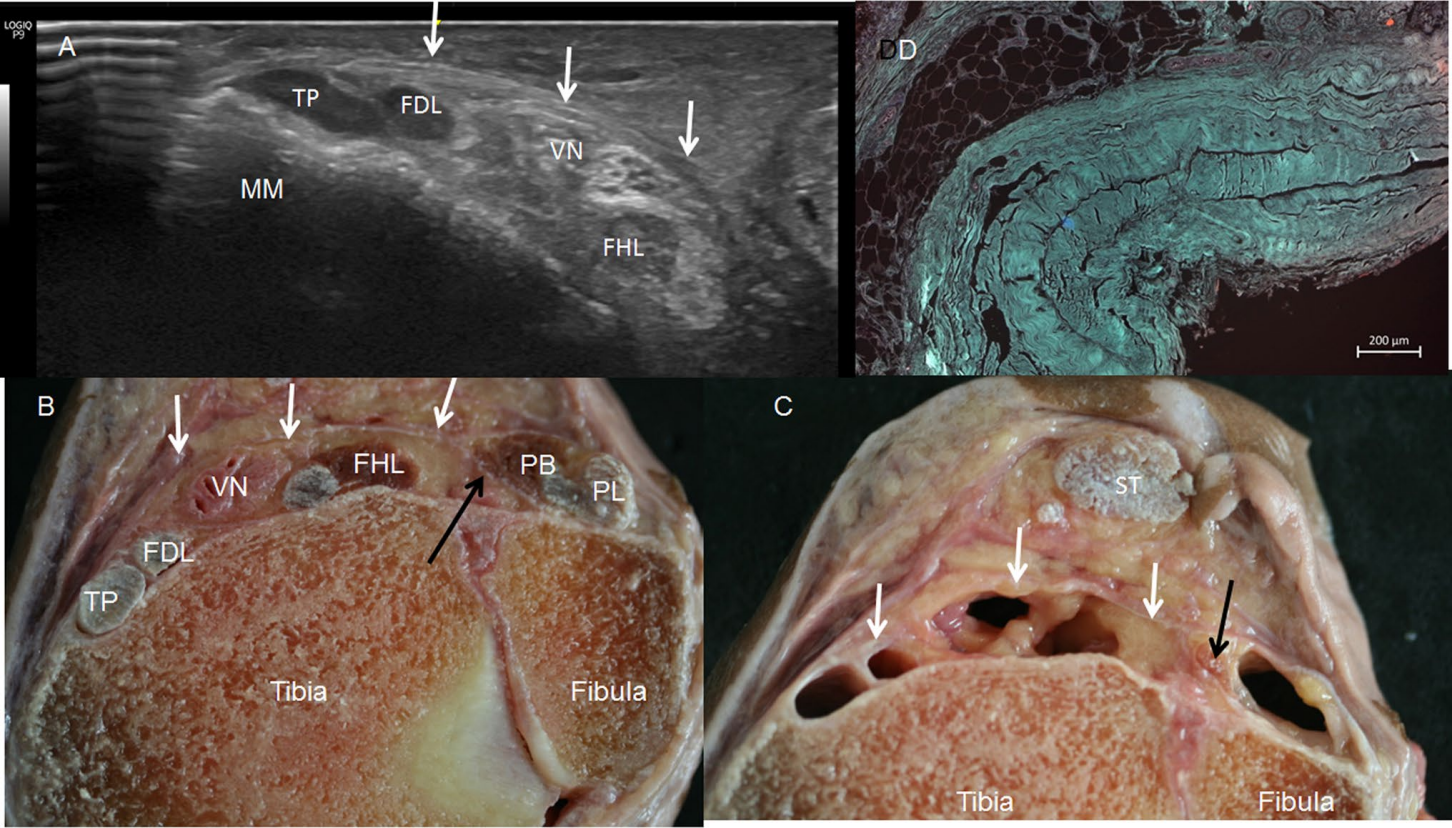
The tendons of the deep posterior muscles of the leg at the medial malleolus.** A** Ultrasound view of the tendons of tibialis posterior (TP), flexor digitorum longus (FDL) muscles, vessels and nerve (VN) and flexor hallucis longus muscle (FHL) just superiorly to the medial malleolus (MM). They are separated from the sural triceps muscle by the septum (white arrow).** B** Transversal anatomical cut of the tendons of the TP, FDL, FHL and VN, fixed by the septum (white arrows) and separated for the peroneus longus (PL) and brevis (PV) muscles for a septum coming from the fascia crural (black arrow)** C** Transversal cut without the tendons, vessels and nerve. It is possible to see the different spaces for each structure.** D** Histological view of the septum with three layers, external and internal are longitudinal and the middle one is transversal

In the posterior compartment, we identified the crural fascia as a hyperechogenic line surrounding the posterior muscles of the leg. This line was fixed, with the medial and lateral malleolus occurring inferiorly. However, another thick hyperechogenic line was observed that began in the inferior middle of the leg (15.75 cm from the head of the fibula and 18 cm from the lateral malleolus), which separated the deep posterior muscles of the posterior compartment as well as the blood vessels and nerves from the triceps surae muscle (Fig. 5A, B) (Table 2). In addition, after the flexor digitorum longus muscle crossed the tibialis posterior muscle, different compartments were observed for the deep posterior tendons of the leg: tibialis posterior, flexor digitorum longus, flexor hallucis longus muscles, and tibialis posterior vessels and tibial nerve (Fig. 6A).

**Table 2:** Descriptive statistics, quantitative variable

Variable	Median (IQR)	Mean (SD) 95%CI mean
Distance HF to LM	33.50 (3)	33.67 (1.74) 32.56–34.77
Exit SFN to HF		
Ultrasonography	21.25 (2)	21.33 (1.05) 20.67–22.0
Anatomy	21.25 (2)	21.42 (1.18) 20.66–22.17
Exit SFN to LM		
Ultrasonography	12.5 (2.389)	12.38 (1.94) 11.14–13.61
Anatomy	12.25 (2)	12.33 (1.93) 11.10–13.56
TA origin at CF to HF		
Ultrasonography	16.25 (3)	15.88 (1.68) 14.81–16.94
Anatomy	15.00 (4)	15.63 (1.69) 14.55–16.70
Origin SPDM to HF		
Ultrasonography	15.75 (3)	15.42 (1.99) 14.15–16.68
Anatomy	15.50 (2)	14.96 (1.54) 13.98–15.94
Origin SPDM to the LM		
Ultrasonography	18.0 (5.13)	18.33 (2.49) 16.75–19.91
Anatomy	18.7 (4.25)	18.71 (2.26) 17.27–20.14

**Fig. 7:**
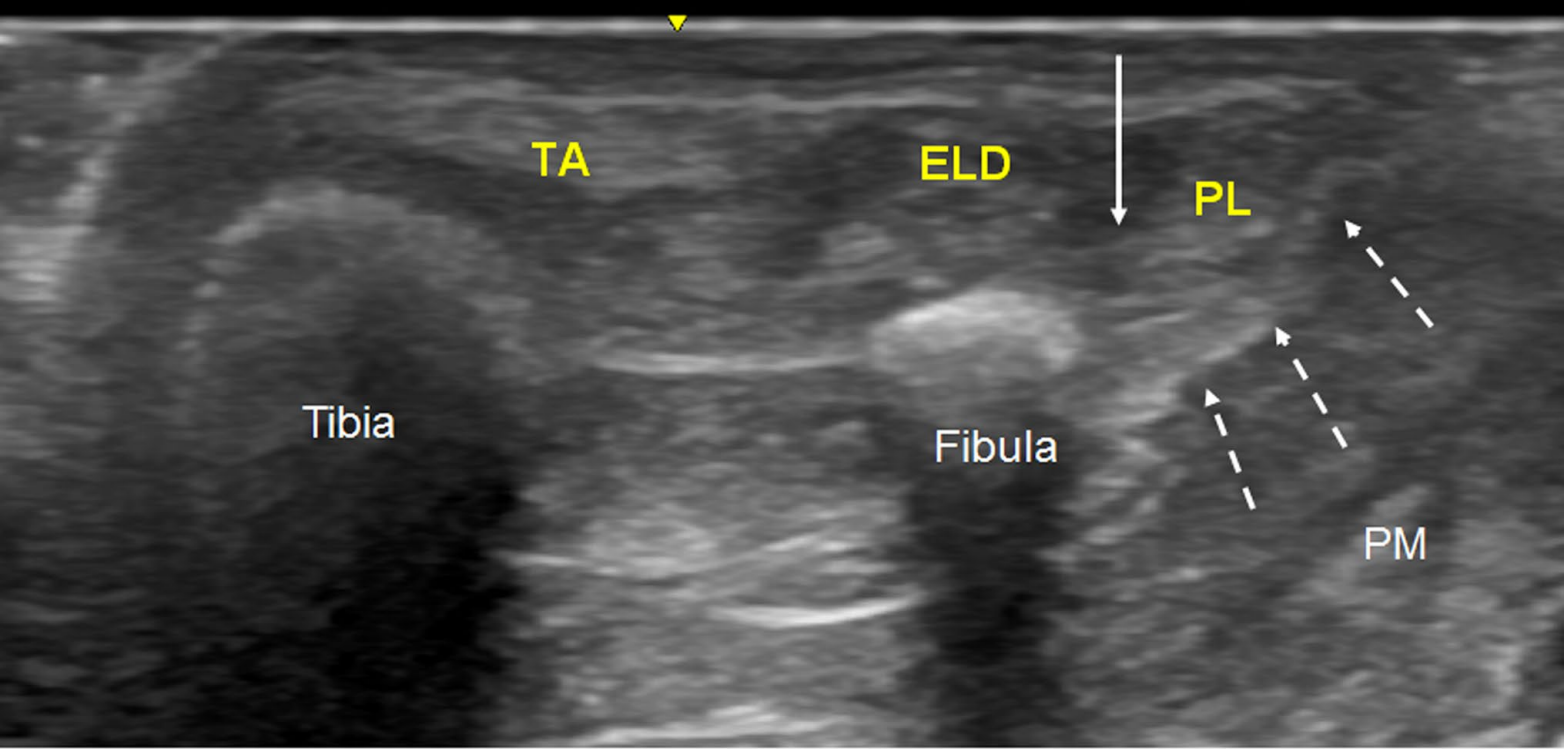
Ultrasound view of the fetus from 29 weeks. There is no septum between tibialis anterior (TA) and extensor digitorum longus (EDL) muscles; however, there is a septum between the EDL and peroneus longus muscle (PL) (white arrow) and PL and posterior compartment (PC) (white broken arrows)

In the fetus, the crural fascia was similar to that of the adult. It formed three compartments, with a deep fascia isolating the deep muscles of the posterior side of the leg, thereby showing the same characteristics as those in the adult; however, the septum between the tibialis anterior muscle and extensor digitorum longus muscle was not observed (Fig. 7).

### Anatomical study

The anatomical study showed that the injected dye remained below the fascia for each of the muscles studied. There was no extravasation of the dye into other muscle compartments in the adults and the fetus, thus demonstrating the independence of the anterior, lateral, and posterior compartments in the leg.

Dissection revealed that the crural fascia in the anterior compartment was fixed superiorly to the tibia and the head of the fibula, and inferiorly to the lateral and medial malleolus. Laterally, the fascia was fixed at the anterior border of the fibula and medially at the anterior border and medial side of the tibia. In the lateral compartment, the fascia was fixed on the fibula, surrounding and isolating the peroneal muscles. In the posterior compartment, superiorly, the fascia was continuous with the fascia lata and was fixed on the tibia and fibula medially and laterally, before reaching the inferior part where it was fixed on the medial and lateral malleolus.

In the anterior compartment, we observed that the crural fascia gave an expansion that continued inferiorly as an aponeurosis that belonged to the TA in 42.8% (5 right and women and 4 left and man) or the EDL in 57.2%. (8 right and 4 left, 7 women and 5 men) of the specimens (Fig. 1C, D). This expansion corresponded to the hyperechogenic line seen as a septum in the ultrasound study that seemed to isolate the TA and EDL. However, in the fetus, no aponeurosis was observed macroscopically for the TA or EDL.

**Fig. 8:**
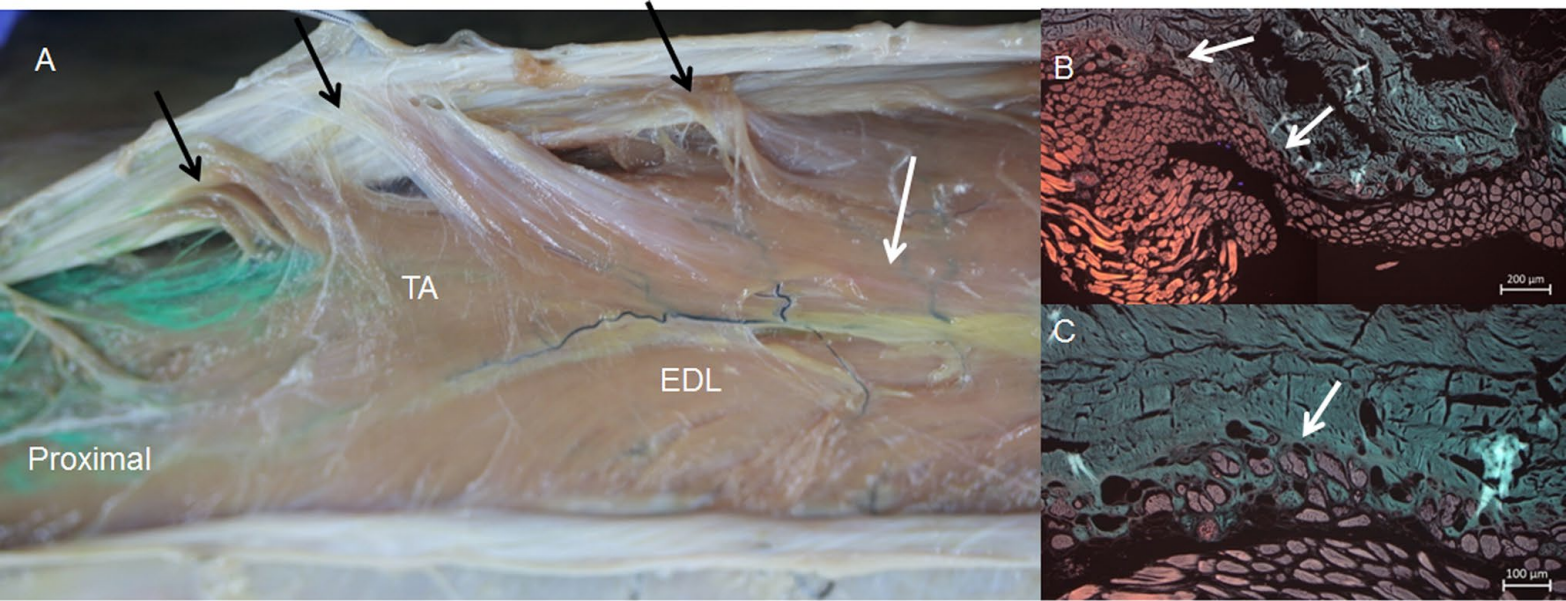
A Anatomic view of the interrupted tibialis anterior muscle origin (TA) at the crural fascia (black arrows) until it arrives to the adipose tissue (white arrow). This adipose tissue separates the TA and extensor digitorum longus muscle (EDL). It is possible to see the green dye that is only at the TA muscle after the ultrasound injection guide. B Histological study shows the fluorescence images of the interrupted origin of the TA seen in red color (white arrows). C Histological view of the deep relation of the muscular fibers (red color) with the fascia (green color) in the point of the origin (white arrows)

We observed that the TA originated at the crural fascia, 15 cm from the head of the fibula and 18.70 cm from the lateral malleolus. This was also noted in the ultrasound study. However, this origin was interrupted (Fig. 8A): just inferior to this region, the ultrasound study showed a hypoechogenic area that corresponded to the adipose tissue separating the TA and EDL (Fig. 2C, D). In the inferior part of the leg, we observed that the tendons of the TA, EDL, and EHL had a deep relationship with the fascia. At this point, the crural fascia formed the superior and inferior retinaculum of the ankle and formed septa to isolate the different tendons (Fig. 3B), as also revealed by the ultrasound study.

**Fig. 9:**
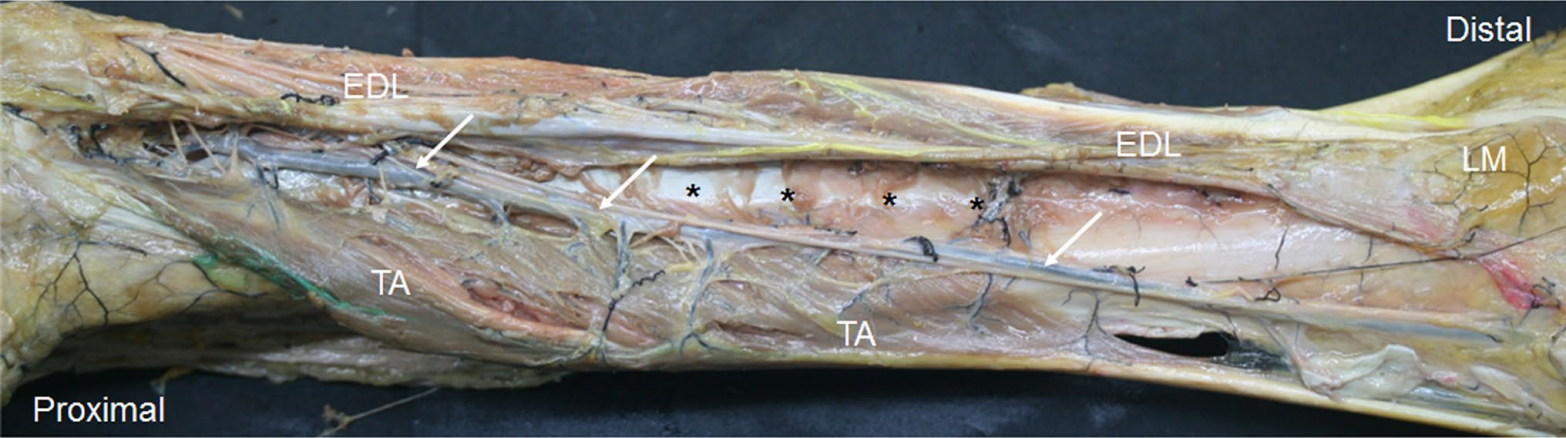
The dissection of the anterior compartment of the leg shows that the tibialis anterior muscle (TA) has a rich vasculature extending transversally from the anterior tibial artery (white arrows), injected by black latex, and located anteriorly to the interosseous membrane (white asterisks). Extensor digitorum longus muscle (EDL) has been away from the TA

A deep dissection of the TA revealed the rich vasculature extending from the anterior tibial artery to the TA (Fig. 9).

In the lateral compartment, the crural fascia was fixed on its lateral surface to the posterior surface of the fibula, isolating the peroneus longus and brevis muscles. The peroneus longus originated directly from the crural fascia before reaching the middle third of the leg. The crural fascia reached the lateral malleolus and there was no septum separating the peroneus longus and brevis muscles superior to the ankle.

**Fig. 10:**
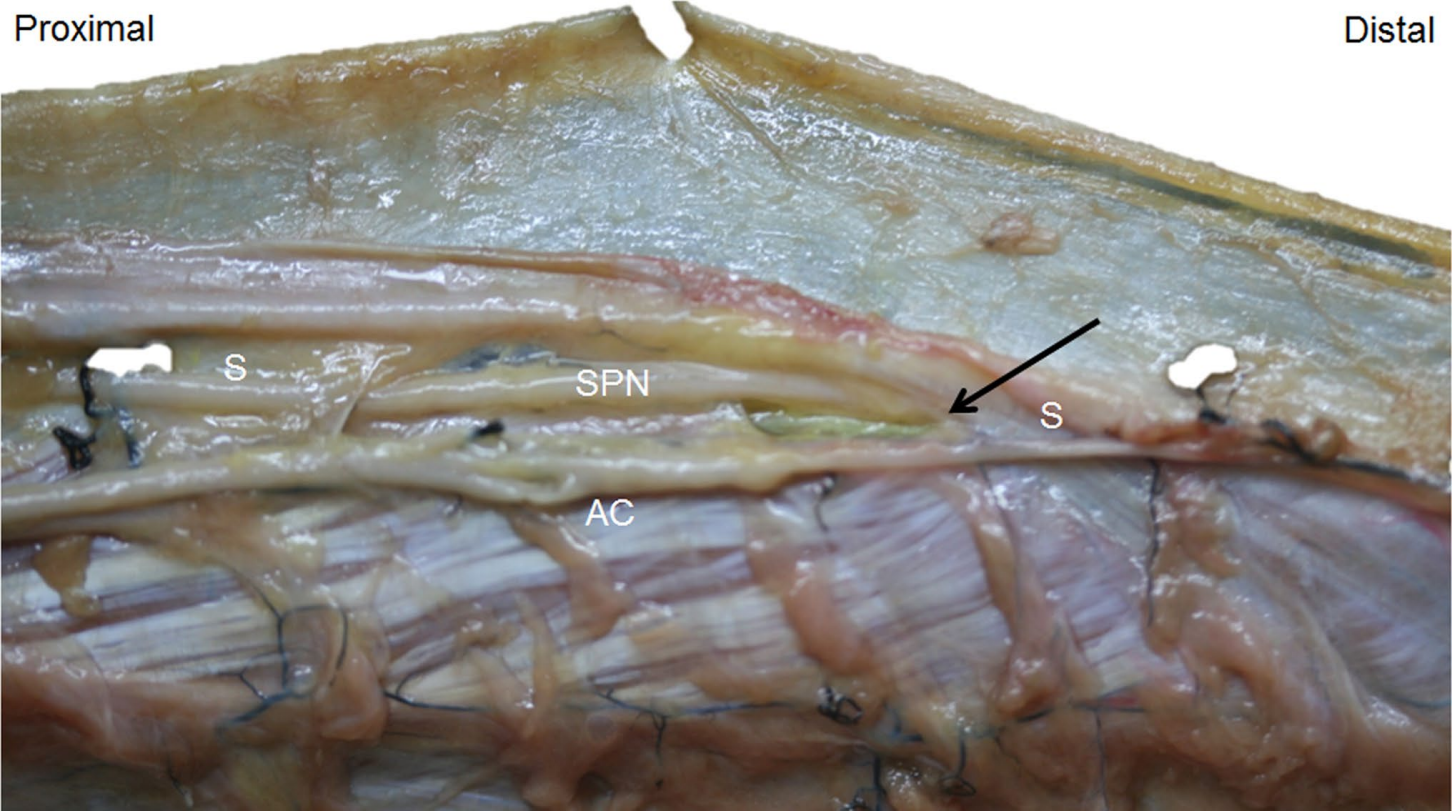
Variation of the superficial peroneal nerve (SPN) that crossed the septum (S) between lateral and anterior compartment to the anterior compartment (AC) to come back to the lateral compartment (black arrow)

We observed a relationship of the superficial fibular nerve with the lateral compartment. The nerve moved from the posterior to the anterior position, crossing the peroneus longus before exiting. It crossed the crural fascia 21.25 cm from the head of the fibula and 12.25 cm from the lateral malleolus. However, the ultrasound showed several variations in its exit. In one case, the nerve had an anterior position in the lateral compartment throughout the pathway. In another case, the nerve crossed the septum between the anterior and lateral compartments and returned to the lateral compartment inferiorly (Fig. 10). And in three other cases, the nerve exited the lateral compartment 3–5 cm before the definitive exit from the crural fascia, but inside a tunnel that the crural fascia had made for it (Fig. 4C, D) as shown by the ultrasound study.

**Fig. 11:**
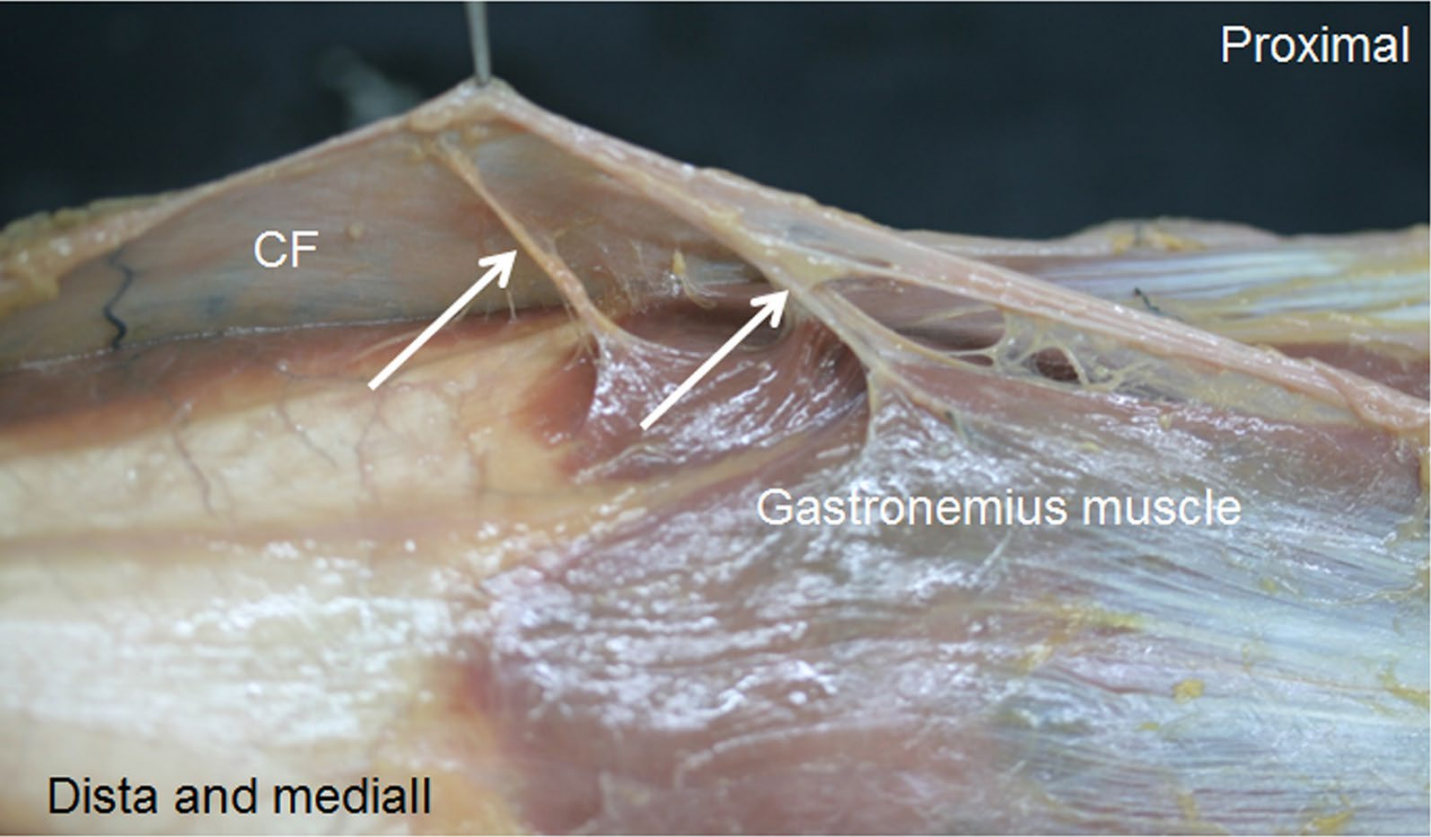
An anatomical view of the relation of the gastrocnemius muscle with the crural fascia (CF) after cutting this fascia. There is no muscular origin of the muscle at the fascia, only fascial connections (white arrows)

In the posterior compartment, the crural fascia surrounded the posterior muscles of the leg. When we cut this fascia along the posterior median line, we observed that the gastrocnemius muscle had not originated from the fascia (Fig. 11) and the fascia followed it laterally and medially before being inserted into the fibula and tibia. Furthermore, inferiorly, there was a fascia covering the deep posterior muscles of the leg, the tibial nerve, and the posterior tibial vessels from the soleus muscle and calcaneal tendon. When the gastrocnemius and soleus muscles were removed (distal to proximal) from the inferior third of the leg, we observed that fascia was a septum to 15.50 cm from the head of the fibula and 18.7 cm from the lateral malleolus. It corresponded to the hyperechogenic septum observed in the ultrasound study. This septum had connective fibers with different directions and patterns, but it enclosed the deep posterior muscles and tendons just before the medial and lateral malleolus where it was fixed (Fig. 5C, D, E). Moreover, a detailed dissection showed that at this point, this fascia followed the crural fascia of the anterior compartment. Just before the medial malleolus and after the sural decussation, this fascia formed different septa for the tibialis posterior and flexor digitorum longus tendons, as well as for the nerve, blood vessels and the flexor hallucis longus tendon just after the sural decussation (the point where the flexor digitorum longus crosses superficially the tibialis anterior muscle) (Fig. 6B, C). We also observed this septum in the fetus but extending superiorly.

### Statistical study

The measurements obtained showed a very good agreement in all the comparisons (Table 2) conducted between the anatomical measures and their ultrasonographic estimations (Table 3, ICC > 0.9). The difference between the anatomical and ultrasound measures was 0.12 cm (95%CI -0.03 to 0.37). The distribution of the differences between the both studies was displayed in a Bland–Altman plot.

**Table 3:** Agreement between anatomical measures and ultrasound estimation

Variable	Coefficient of correlation intraclass [CCI] (IC 95% CCI)	ANOVA F(P) mixed model, type absolute
Exit SFN to HF		
Ultrasonography	0.917 (0.742–0.975)	21.828 (0.000007)
Anatomy		
Exit SFN to LM		
Ultrasonography	0.964 (0.88–0.989)	50.239 (8.838 E-8)
Anatomy		
TA origin at CF to HF		
Ultrasonography	0.853 (0.583–0.955)	12.541 (0.000108)
Anatomy		
Origin SPDM to HF		
Ultrasonography	0.901 (0.624–0.972)	25.637 (0.000003)
Anatomy		
Origin SPDM to the LM		
Ultrasonography	0.95 (0.82–0.9)	43.73 (1.8462E-7)
Anatomy		

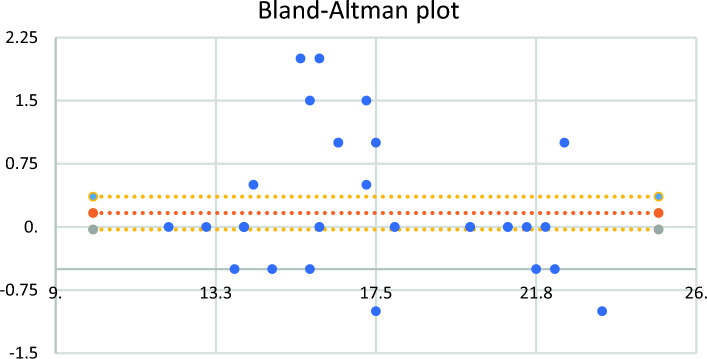

### Histological study

**Fig. 12:**
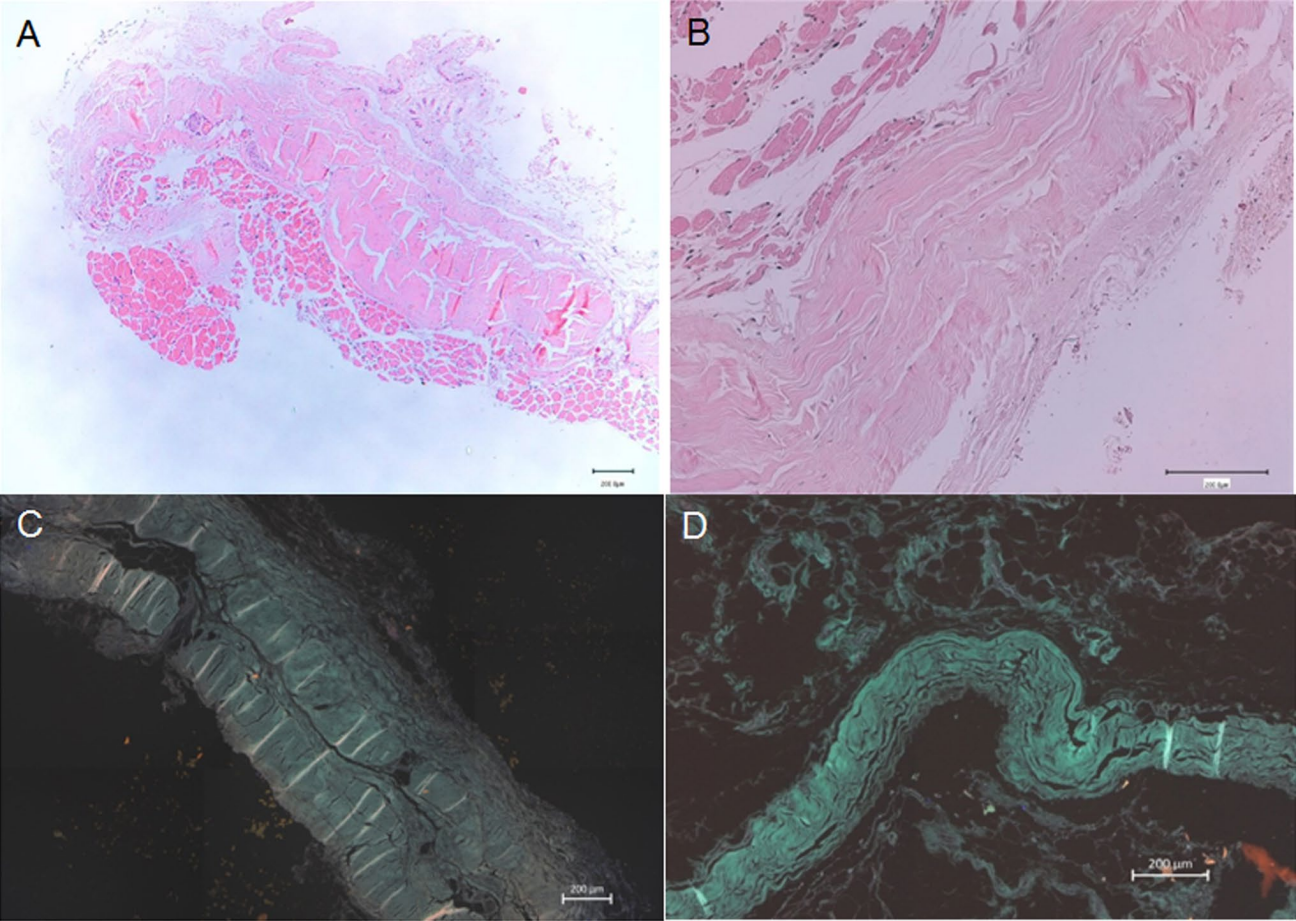
Histological study of the fascia of the anterior compartment by haematoxylin–eosin stain** A** at the superior level** B** at the middle level.** C** and** D** Fascia at the posterior compartment observed by the fluorescence microscopy images** C** at the superior third of the leg.** D** at the inferior third of the leg. In the superior part, the fascia has more transversal layers and in the inferior part, the layers are all longitudinal

All the samples showed the organization of the connective tissue into two or three different layers depending on the regional areas. Moreover, we found some differences between them. We observed that the crural fascia, at the anterior compartment in the superior part of the leg, was much thicker (0.840 mm) than in the inferior part (0.198 mm) before it reached the superior retinaculum. The morphology of the crural fascia at the posterior compartment was very similar, with a great thickness, at the superior side than at the inferior side, (0.841 mm vs 0.231 mm). The disposition of the layers was transversal and longitudinal at the superior part (Fig. 12A, B) and longitudinal at the inferior part are (Fig. 12C, D). In the superior retinaculum, the crural fascia showed differences just above the TA, presenting several parallel layers of dense connective tissue and a thickness of 0.567 mm. However, above the EDL, the dense connective tissue changed morphology and became thicker, increasing to 0.356 mm (Fig. 3C, D). Also at the anterior compartment, and correspondingly, as observed in the macroscopic anatomical study, we noted a relationship between the fascia and the muscle fibers of the TA. In some places, the muscle fibers were inside the fascia; while in others, the muscle fibers showed no relationship with the fascia (Fig. 8B, C).

Finally, the fascia covering the deep posterior muscles of the leg had a thickness of 0.689 mm (Fig. 6D).

## Discussion

The study of the crural fascia revealed a strong relationship between this fascia and the muscle fibers of the TA. The TA makes up the majority of the muscle volume in the leg anterior compartment (59.7%) [14]. Moreover, the origin of the TA in this fascia, with attachments interrupted, 16.25 cm from the head of the fibula, can be identified by ultrasound through the longitudinal orientation of the fascial fibers. Distally to this distance, the presence of a hypoechogenic area between the TA and EDL can be used to avoid the cutting the fibers of the origin of the TA on the crural fascia. We propose this as a new approach, during a fasciotomy, in the surgical treatment of compartment syndrome and as a way to resolve the frequent ischemia of the TA [8]. This procedure has not been reported before. Furthermore, it would prevent the development of chronic compartmental syndrome by reducing the amount of muscle fibers unintentionally damaged during surgery [8].

Distally, just above the ankle, the crural fascia forms the extensor retinaculum which isolates the tendons of the TA, EDL and EHL. We postulate that this superior retinaculum is important for the functions of these muscles in the foot. It forms a special canal for each tendon. Furthermore, the histological differences observed among the tendons just above the TA and EDL may explain why that the tendon of the TA is reinforced and more firmly attached for a better pulley and dorsal flexion of the foot.

The involvement of the anterior compartment is usually accompanied by the involvement of the lateral compartment in chronic [7] and acute compartment syndrome [27]. This may lead to damage of the superficial fibular nerve. The anatomical study showed that the nerve exited 21.25 cm from the head of the fibula and 12.25 cm from the lateral malleolus. Our results are similar to those of other articles that have measured the distance between the exit of the superficial fibular nerve from the crural fascia and of the lateral malleolus apex. For example, one study reported a distance of 10.33 cm for intraseptal cases and 11.28 cm for extraseptal cases [26], while another study reported 11.36 cm ± 4.39 cm [20]. However, as in those studies, we observed a great variability in the number of nerve divisions [28] and their trajectory [20]. The frequency of these variations is similar to that reported by other authors as 23% [10], although some studies have found only 8.3% [1]. For this reason, in agreement with other authors [2, 20], we believe that the use of ultrasound should be mandatory when performing a fasciotomy to avoid any iatrogenic damage to the superficial fibular nerve.

Regarding the posterior compartment, also in agreement with the previous studies, we observed that the crural fascia surrounds the posterior muscles, but neither of the heads of the gastrocnemius is attached to the fascia. These anatomical characteristics are of special importance for the muscles that form the superficial compartment, allowing them to move independent of their fascia during powerful contractions in weight-bearing activities [4]. In this compartment, the ultrasonographic study identified the fascia that covers the deep posterior muscles as a hyperechogenic and proved in the anatomical dissection. It forms a broad fascial compartment that enclosed and attached the flexor digitorum longus, the tibialis posterior, the flexor hallucis longus, muscles, the tibial nerve, and the posterior tibial vessels, and separated them from the soleus muscle. It is reinforced by some transversal bands which may explain why it is necessary to reopen and remove strips of this fascial tissue in the surgical treatment of recurrent chronic exertional compartment syndrome [9, 25]. Thus, this information may lead to a re-exploration of the three deep muscles, avoid complications [29, 30], and be helpful in establishing a standard number of required incisions [11]. However, surgery in this compartment has low success rate [11, 25] because there is a small space between the fascia and the muscles, increasing the possibility of damaging the muscles as well as the blood vessels and nerve that are associated with these muscles [29, 30]. Nevertheless, the ability to identify the fascia by ultrasound, as we were able to do, could improve the gold standard approach of opening the fascia and accessing the superficial and deep compartments. More studies are now required to improve the efficiency of the technique [25, 29].

The histological study shows that this septum was composed of dense connective tissue arranged into several layers. However, we cannot compare these findings with those of other studies. This fascia was thicker in the inferior area of the leg before becoming fixed on the medial and lateral malleolus.

Regarding the histological study, our findings of the morphometric evaluation of the fascia were similar to those of other studies, which have reported a mean thickness of 1 mm. [23]. However, we observed some differences in the thickness of the fascia at different points in the leg. For example, in the superior part of the anterior and posterior compartment, the fascia was thicker than in the inferior part of the anterior and posterior compartment. Moreover, this difference in thickness was larger in the posterior part of the leg, possibly due to the larger numbers of muscle fibers of the TA and EDL. In the dorsal superior retinaculum, the thickness was greater over the TA than the EDL, even with the different morphology of the superior retinaculum. This might be because the tendon of the TA has to be better fixed to allow the foot to perform its movements. Curiously, the fascia covering the deep posterior muscles of the leg, presented the highest values for thickness of all those obtained in this study.

In the fetus, we confirmed the presence of a deep fascia surrounding the leg. It has been previously reported that the fascia is present from week 21 of human development [5]. Moreover, we found that the fascia forms three different compartments in the leg at 29 weeks of human development, as in the adult. It is perfectly differentiated for muscle movement in the womb, possibly facilitating the configuration of the leg fasciae. However, we did not observe the presence of an aponeurosis separating the TA and EDL. Furthermore, the posterior fascia of the deep posterior muscles was thicker in the fetus than in the adult and tended to decrease in thickness, as demonstrated in a previous study [18].

## Conclusions

This study broadens our knowledge of the ultrasound, anatomical, and histological characteristics of the crural fascia. This information may help to prevent damage to structures (such as the superficial fibular nerve, the vascular area of the deep posterior compartment muscles, and the TA muscle fibers) during the surgical treatment of acute and chronic compartment syndrome. Our findings allow a better identification of these structures, facilitating the diagnosis of any pathology in the area, and has several potential clinical and therapeutic applications.

## Data Availability

We declare that there is no problem to access the datasets used.

## Abbreviations

TA: Tibialis anterior muscle
EDL: Extensor digitorum longus muscle
EHL: Extensor hallucis longus muscle
PL: Peroneus longus muscle
SFN: Superficial fibular nerve
IQR: Interquartile range
ICC: Coefficient intraclass correlation
